# The Effect of Fractionation during the Vacuum Deposition of Stabilized Amorphous Selenium Alloy Photoconductors on the Overall Charge Collection Efficiency

**DOI:** 10.3390/s22197128

**Published:** 2022-09-20

**Authors:** Safa Kasap

**Affiliations:** Department of Electrical and Computer Engineering, University of Saskatchewan, Saskatoon, SK S7N 5A9, Canada; safa.kasap@usask.ca

**Keywords:** stabilized amorphous selenium, fractionation, X-ray sensitivity, photoconductivity, charge collection efficiency

## Abstract

The general fabrication process for stabilized amorphous selenium (a-Se) detectors is vacuum deposition. The evaporant alloy is typically selenium alloyed with 0.3–0.5%As to stabilize it against crystallization. During the evaporation, fractionation leads to the formation of a deposited film that is rich in As near the surface and rich in Se near the substrate. The As content is invariably not uniform across the film thickness. This paper examines the effect of non-uniform As content on the charge collection efficiency (CE). The model for the actual CE calculation is based on the generalized CE equation under small signals; it involves the integration of the reciprocal range-field product (the schubweg) and the photogeneration profile. The data for the model input were extracted from the literature on the dependence of charge carrier drift mobilities and lifetimes on the As content in a-Se_1−*x*_As*_x_* alloys to generate the spatial variation of hole and electron ranges across the photoconductor film. This range variation is then used to calculate the actual CE in the integral equation as a function of the applied field. The carrier ranges corresponding to the average composition in the film are also used in the standard CE equation under uniform ranges to examine whether one can simply use the average As content to calculate the CE. The standard equation is also used with ranges from the spatial average and average inverse. Errors are then compared and quantified from the use of various averages. The particular choice for averaging depends on the polarity of the radiation-receiving electrode and the spatial variation of the carrier ranges.

## 1. Introduction and Objectives

Stabilized amorphous selenium (a-Se) is widely used in commercial mammographic imaging detectors as an X-ray photoconductor [1]. The properties of a-Se have recently been reviewed in [2] (see also references therein). The general fabrication process for a-Se detectors involves the vacuum deposition of the photoconductive film onto an imaging substrate held at a temperature usually above the glass transition temperature of the source alloy, but below the crystallization temperature. The film thickness is roughly 200 μm for mammographic detectors, but thicknesses around 1000 μm have been used for fluoroscopic detectors. The evaporant alloy is typically pure selenium alloyed with 0.3–0.5%As in the form of vitreous solid pieces, often small pellets or shots. The small amount of As-alloying stabilizes a-Se against crystallization. During the evaporation, fractionation leads to a deposited film that is rich in As near the surface and rich in Se near the substrate [3,4,5]. The As content is therefore not uniform throughout the film thickness. It is possible to avoid excess As near the surface and As deficiency near the substrate by suitably shuttering the boat or the substrate, but this leads to wasted source material. It is easier to turn off the evaporation near the end and thus avoid a large As concentration at the surface.

The As-fractionation during the vacuum deposition of vitreous pellets from a directly heated metal boat is known to depend on various conditions, most notably: (1) the composition of the evaporant alloy; the alloy is assumed to be uniform in composition. (2) The rate of evaporation, which depends on the melt temperature. (3) The temperature nonuniformity in the melt. (4) The temperature–time profile of the boat during the deposition, and hence the evaporation rate history. (5) The volume of the melt in the boat and the boat geometry. (6) The aging of the source material. Aging changes the structure of a-Se alloys and prolonged aging can lead to partial crystallization of the source material. (7) The vacuum chamber pressure. (8) The configuration of the vacuum chamber, e.g., the substrate’s location with respect to the source. (9) The degree of melt depletion in the boat. (10) The film thickness.

An obvious question is then “What is the effect of fractionation during the a-Se deposition on the X-ray sensitivity?”. This paper examines the effect of non-uniform As content on the charge collection efficiency (CE), which is an important component of X-ray sensitivity. The methodology for evaluating the worst-case performance is based on the following steps:

First, a typical variation in the As content throughout the film thickness is considered from data reported in the literature. Second, using the data on the As content vs. distance into film, the spatial variation of the hole and electron ranges (the drift mobility and lifetime products) is constructed. Third, using the carrier range profiles, the charge carrier CE is calculated based on the formulation summarized in [6]. This CE is compared with the corresponding case of a uniform composition a-Se film, assuming an average As content throughout the film. The CE is calculated as a function of the applied electric field; small signals are assumed. In addition, an “average” definition based on spatial averaging (SA) and the average inverse (AI) are also considered in the standard CE equation. Although the semiconductor in this work is a-Se, charge CE is an important metric of photoconductive detectors, especially those used for high energy radiation detection. Some of the conclusions from this work are likely to be generally useful in understanding the CE of detectors based on different photoconductive materials.

Both positive and negative biases on the radiation receiving electrodes are considered. The practical p-i-n a-Se detector also has very thin blocking layers (the n- and p-layers) next to the bias electrodes, which are not considered in this work. Put differently, only the i-layer is considered, because the photoconductor action takes place in this layer and it is the thickest layer in the detector structure. A hole entering the thin p-layer recombines with a trapped electron in this layer, which is replenished by an electron injected from the negative electrode next to the p-layer. This is equivalent to stating that the hole that arrives in the p-layer becomes collected. Similarly, an electron arriving in the n-layer recombines with a trapped hole in this layer. The lost hole is replaced by injection from the positive electrode next to the n-layer. Thus, the electron effectively becomes collected. Section 2 formulates the present model and the three steps that have been used. Section 3 provides the results and discusses their impact. A summary and conclusions are given in Section 4.

**Figure 1:**
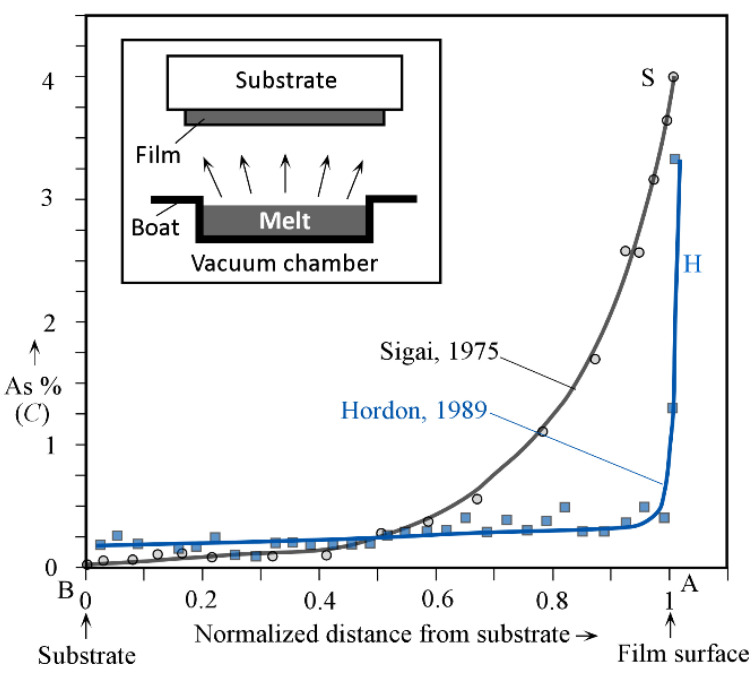
As concentration (%) as a function of normalized thickness *x*/*L* across the film thickness of two evaporated a-Se films. Data have been extracted, normalized, combined, and replotted from Sigai 1975 [4] (sample S), and Hordon 1989 [5] (sample H). The As content in the source material is 0.50% for S and 0.34% for H. Both films were approximately 60 μm in thickness. (A and B correspond to the film surface and substrate respectiveley).

Figure 1 shows the variation of the As content across two typical vacuum-deposited a-Se films, as reported in two separate works [4,5]. (The compositions are typically quoted in wt.% in materials science and, since the relative atomic masses of Se and As are 78.96 and 74.92, respectively, 1.00 wt% As corresponds to 1.05 at.% As.) The distance from the substrate has been normalized to the total thickness *L*. In both cases, the film thickness was approximately 60 μm. An electron microprobe (probably with EDX) was used for sample S and the beam size was ~2 μm. No information was given for sample H. Note that the labels A and B refer to the radiation-receiving surface and the substrate surface of the a-Se film, respectively.

Sample S in Figure 1 [4] was evaporated from a source composition of 0.5%As, whereas H used 0.34% As as its source material. It is clear that, in the case of S, the sample possesses extensive fractionation. The average As content in S is 0.69% and the standard deviation is 0.90%. The As content is almost nil right next to the substrate and increases monotonically and sharply with the evaporation time, i.e., the distance into the film. The As content on the surface is over 4%. Even higher surface As content has been reported in various vacuum-coated a-Se films. Such a large variation would render this film an inferior photoconductor, because once the As content reaches ~1% and exceeds it, the hole transport deteriorates rapidly. Clearly, the region of *x*/*L* from 0.8 to 1 would trap holes and prevent these from being collected, leading to a reduction in the CE.

In the case of sample H, there is an approximately linear increase in the As content with distance, but the As content at the surface is over 3%. However, this high As surface region is very thin (much less than 1% of *L*) and its effect on the CE can be neglected even if holes become trapped on the “surface”. The collected charge depends on the integration of the photocurrent, which is dominated by the normalized thickness from *x*/*L* from 0 to 0.99. The As variation in sample H is considered to be typical for a practical a-Se-based photoconductive layer: an optimum that balances fractionation in the film (surface excluded) with the deposition rate and waste of source material. Thus, samples S and H refer to near-worst-case and near-optimum-case As variation in a-Se photoconductors.

## 2. Formulation of the Model

### 2.1. Arsenic Content Profile

**Figure 2:**
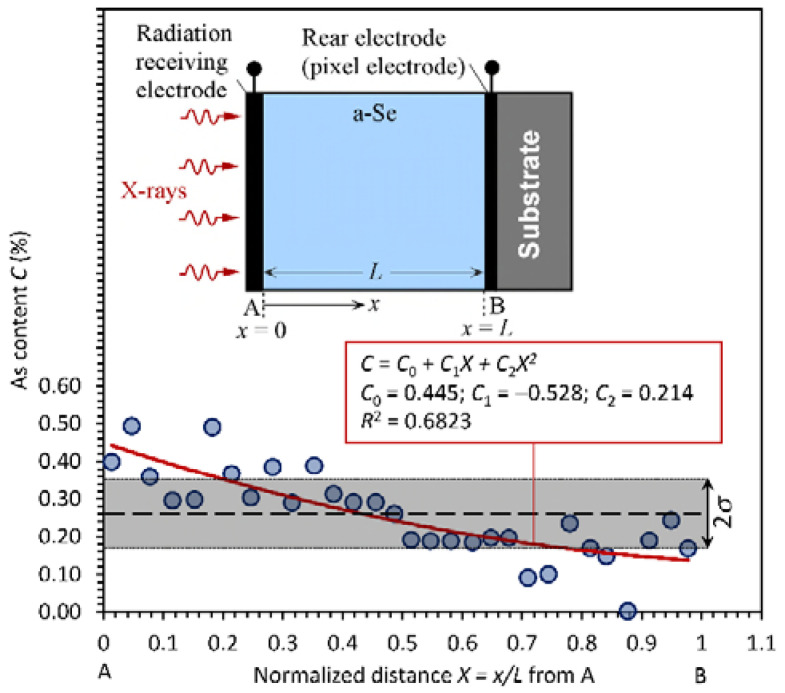
As content variation in sample H in Figure 1 as a function of the normalized distance from the radiation-receiving electrode A. The red curve is the best second-order polynomial fit. The inset shows the device geometry. (Data extracted from [5], normalized and reanalyzed to plot the As content from the radiation-receiving side, A, toward the substrate, B).

**Figure 3:**
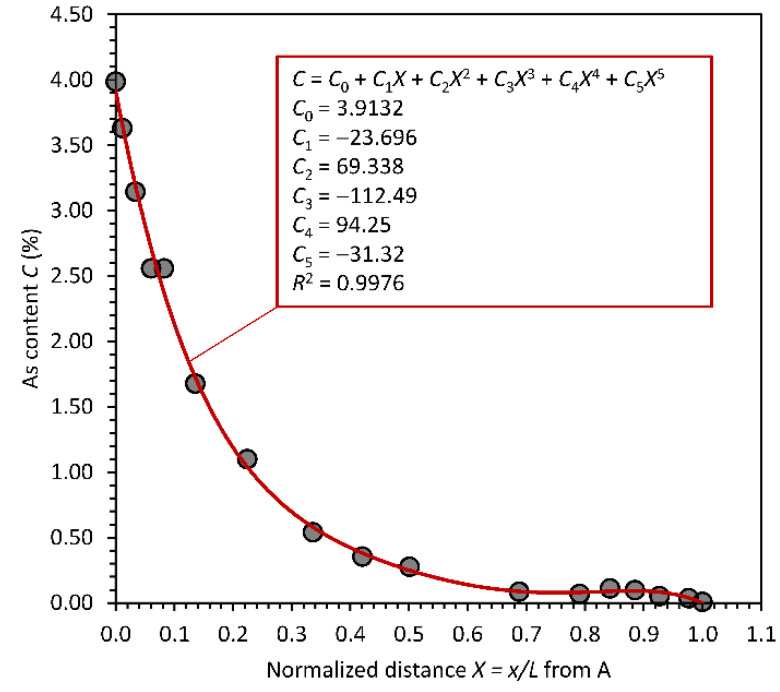
As content variation in sample S in Figure 1 as a function of the normalized distance from the radiation-receiving electrode. The best polynomial (fifth order) is also shown (Data extracted from [4], normalized and reanalyzed to plot the As content from the radiation-receiving side).

The first step in modeling the effect of fractionation is to model the As content *C* variation with normalized distance *X* = *x*/*L* into film. Figure 2 shows the plot of Hordon’s data (sample H in Figure 1) from the radiation-receiving side, with distance normalized to the sample thickness *L*. The best representation of the data is a second-order polynomial, since high-order polynomials generate oscillations in *C* vs. *X* which are not physically possible. The average As content is found by integrating the best fit polynomial *C*(*X*) from *X* = 0 to 1, which gives $\bar{C}$ = 0.26%, a value lower than the bulk value in the evaporant (0.34%). The root mean square deviation (RMSD) is found by integrating ${\left[C\left(X\right)-\bar{C}\right]}^{2}$ and then square rooting it. Its value is 0.092%. It should be apparent that Hordon’s data in Figure 2 reveal a parabolic and monotonic decrease in *C* with *X*. In a similar fashion, Sigai’s data are replotted in Figure 3, where it should be apparent that the best fit polynomial is a fifth order up to the term *X*^5^. Table 1 summarizes the expected characteristics of fractionation in both samples H and S: a near-optimum-case commercial-type a-Se layer, and a near-worst-case sample. The As content *C* = *C*(*X*) as a function of *X* is represented by a polynomial of the type,

(1) $$C\left(X\right)=C_{0}+C_{1}X+C_{2}X^{2}+C_{3}X^{3}+C_{4}X^{4}+C_{5}X^{5};\;X=x/L\;$$

The *C*-coefficients for both samples are listed in the figures and also in Table 1. The data for sample H have a larger scatter than those for sample S. While an excellent fifth-order polynomial can be fitted to sample S, for sample H, a high-order polynomial greater than two generates unrealistic oscillations in *C* with *X*, as mentioned above, and has been excluded. It is also possible to fit a linear decrease in *C* with *X*, as is apparent from Table 1.

### 2.2. Carrier Range Dependence on As Content

The dependence of the hole and electron ranges, *r_h_* and *r_e_*, on the As content has been studied by several authors as reviewed in [2]. In nearly all cases, the quoted As content is for the bulk, that is, the composition of the source material. The bulk As content, which is uniform in the vitreous a-Se pellets, can be converted to the average As content in the film by noting that the average As film content is roughly a factor of 0.7 of that in the bulk. Figure 2 and the data in Table 1 for the film H make the film-to-bulk As content ratio 0.257/0.34 or 0.756. The average As content in the film when the bulk is 6.0% is 4.0%, i.e., a ratio of 0.667 in [7]. An average of 0.71 was used to obtain the average film content for the analysis of the dependence of the carrier ranges on the As content in the film. The carrier range determinations typically involve time-of-flight (TOF), interrupted field time-of-flight (IFTOF), and first xerographic residual voltage measurements which have uncertainties and errors in the range of 10–15% [2]. The composition measurements also have uncertainties (as apparent in Figure 2). Consequently, a more accurate bulk-to-film composition conversion is not expected to improve the accuracy of the model or affect quantitative conclusions.

**Figure 4:**
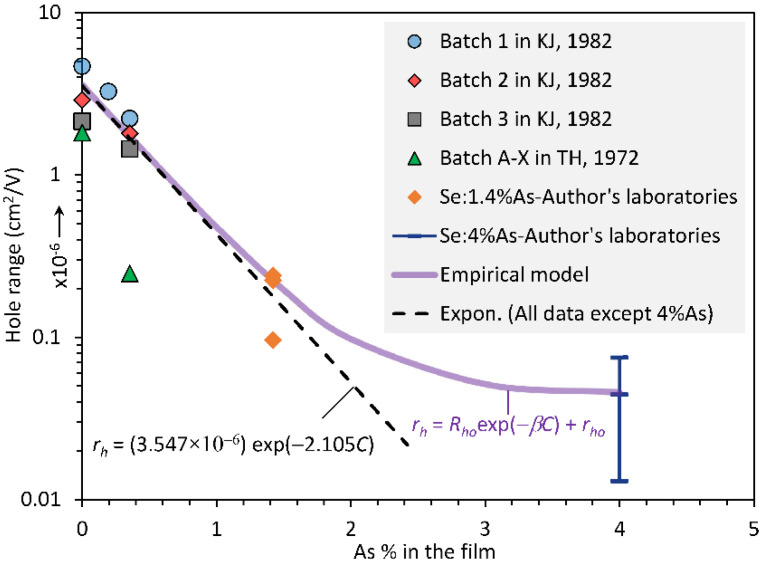
A semilogarithmic plot of the dependence of the hole range *r_h_* on the As content *C* in the film. The initial hole lifetime has batch-to-batch variations. Data were extracted, reanalyzed, converted to the average As content in the film, and then replotted. KJ, 1982, and TH, 1972, are [9,10], respectively. Note: *R_ho_* = 3.547 × 10^−^^6^ cm^2^ V^−1^ is a preexponential constant, *β* = 2.105 percent^−1^.

Figure 4 shows a semilogarithmic plot of the dependence of the hole range *r_h_* = *μ_h_τ_h_*, where *μ_h_*, and *τ_h_* are the hole drift mobility and lifetime, respectively, on the As content in the film as reported in a number of studies. In all cases, *r_h_* was observed to decrease with small amounts of As alloying. The *r_h_* initially appears to decrease almost linearly, but then flattens at high As content around ~4%. The latter observation is in line with the work in [8], where the hole response was shown to drop and flatten around 4%As. There is a large variation in the hole range in pure a-Se, which depends on the source of the material and batch [2,9,10]. An empirical equation *r_h_* = *f*(*C*) is needed to account for the experiments in Figure 4 so that this function can be used in the CE calculations. The following empirical equation, shown in Figure 4, was found to represent the overall behavior:

(2) $$r_{h}\left(C\right)=R_{ho}\exp\left(-\beta C\right)+r_{ho}\;$$

where *R_ho_* = 3.55 × 10^−6^ cm^2^ V^−1^ is a preexponential constant, *β* (=2.105 percent^−1^) is a characteristic constant that describes the rate of decay of *r_h_* with *C*, and *r_ho_* = 0.045 × 10^−6^ cm^2^ V^−1^ represents the plateau region of the data. Equation (2) is plotted in Figure 4. With Equations (1) and (2), we can represent the variation in *r_h_* across the film thickness for both samples H and S, i.e., *r_h_* = *r_h_*(*x*). It is important to mention that the hole range *r_h_* depends on the substrate temperature during deposition [2]. It is assumed that the a-Se film that is to be used as a photoconductive layer has been deposited at a substrate temperature above the glass transition temperature of the source alloy.

**Figure 5:**
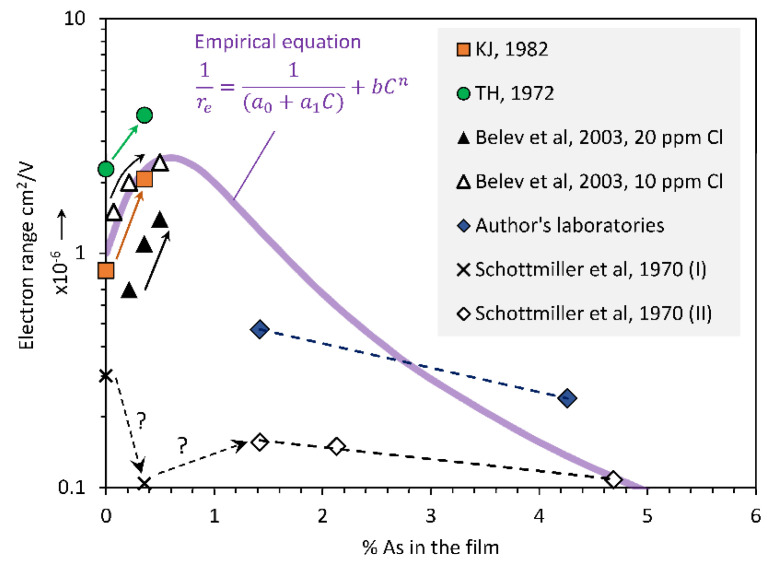
A semilogarithmic plot of the dependence of the electron range *r_e_* on the As content *C* in the film. The initial electron lifetime has batch-to-batch variations. Data were extracted, reanalyzed, converted to the average As content in the film, and then replotted. KJ, 1982, and TH, 1972, are [9,10], respectively, Schottmiller et al., 1970, is [8], Belev, 2003, is [16]. Note: *a*_0_ = 1.0 × 10^−6^ cm^2^ V^−1^, *a*_1_ = 4.0 × 10^−6^ cm^2^ V^−1^ percent^−1^, *b* = 0.3 × 10^6^ V cm^−2^ percent^−2.2^, and *n* = 2.2.

The dependence of the electron transport on the As content has been studied in a number of previous works. It is well known that the electron transport in a-Se tends to vary by a large extent between batches and suppliers [2,3,10,11,12], which is probably due to its sensitivity to small amounts of impurities in the ppm range. For example, it is well known that ppm amounts of halogens in a-Se can drastically reduce the electron transport but enhance the hole transport. In addition, unlike the hole range, the electron range does not depend on the substrate temperature [13]. Experiments indicate that, initially, the electron range increases with the As content but then falls sharply. Early work suggested that electron transport is absent when the As content exceeds roughly 8%, since the authors in [14] did not extend their study of a-Se_1−*x*_As*_x_* alloys beyond 8%. There have been no reports of electron transport in a-Se alloys with As beyond 8%, and it is totally absent in a-As_2_Se_3_ films. Furthermore, there is a sudden increase in the conductivity activation energy around 9% As, as reported in [15]. Figure 5 shows a semilogarithmic plot of the dependence of the electron range *r_e_* on the As content *C*. The three works [9,10,16] clearly point to an increase in *r_e_* with small amounts of As-alloying, roughly up to ~0.7%As. In the latter works, the addition of As was from the same supplier and batch. Once the As content is greater than ~0.7%, there is a sharp fall in the electron range with the As-content, with no measurable electron transport beyond ~8%.

One of the main problems in extracting the carrier range from the shape of the TOF photocurrent is that the decay in the photocurrent may not be entirely due to trapping. With a spatially varying As content, there will be a spatially varying electron mobility. Since the photocurrent *i*_ph_(*t*) is, at any instant, proportional the drift mobility *μ_e_* at the location of the drifting carrier package, and since *μ_e_* drops exponentially with the As content, it is apparent that the shape of *i*_ph_(*t*) cannot simply be ascribed to deep trapping. IFTOF measurements are more accurate, since the carrier packet’s drift is halted for a certain time period, the interruption time *t_i_*, and the fractional change in the photocurrent is measured as a function of *t_i_* as described in [17]. An examination of the electron range data in Figure 5 reveals that *r_e_* initially increases with *C*, peaks around ~0.7%, and then drops sharply with the As content. One possible empirical expression for *r_e_* is:

(3) $$r_{e}\left(C\right)={\left[\frac{1}{a_{0}+a_{1}C}+bC^{n}\right]}^{-1}$$

where, in this work, *a*_0_ = 1.0 × 10^−6^ cm^2^ V^−1^, *a*_1_ = 4.0 × 10^−6^ cm^2^ V^−1^ percent^−1^, *b* = 0.3 × 10^6^ V cm^−2^ percent^−2.2^, and *n* = 2.2. Equation (3) is plotted in Figure 5, where it can be seen that it captures the initial rise well, and then provides a generic decay to *r_e_*, roughly as a function of *C*^−2.2^. One can easily generate other empirical equations that can describe the observations in a general way. What is important is the general shape (that is, the rise), the position of the peak and fall, because the CE is controlled by the carrier schubweg, the product of the carrier range, and the field. The schubweg is defined as the average distance drifted by a carrier before it is trapped and removed from the transport band. If *μ_e_* and *μ_h_* are the electron and hole drift mobilities, respectively, and *τ_e_* and *τ_h_* are the electron and hole lifetimes, respectively, the electron and hole schubwegs are defined by *s_e_* = *μ_e_τ_e_E* = *r_e_E* and *s_h_* = *μ_h_τ_h_E* = *r_h_E*, respectively. It is apparent that the functional form of the spatial dependence of the carrier range is of interest since the absolute value can be shifted by changing the field.

### 2.3. Spatial Dependence of Carrier Ranges

**Figure 6:**
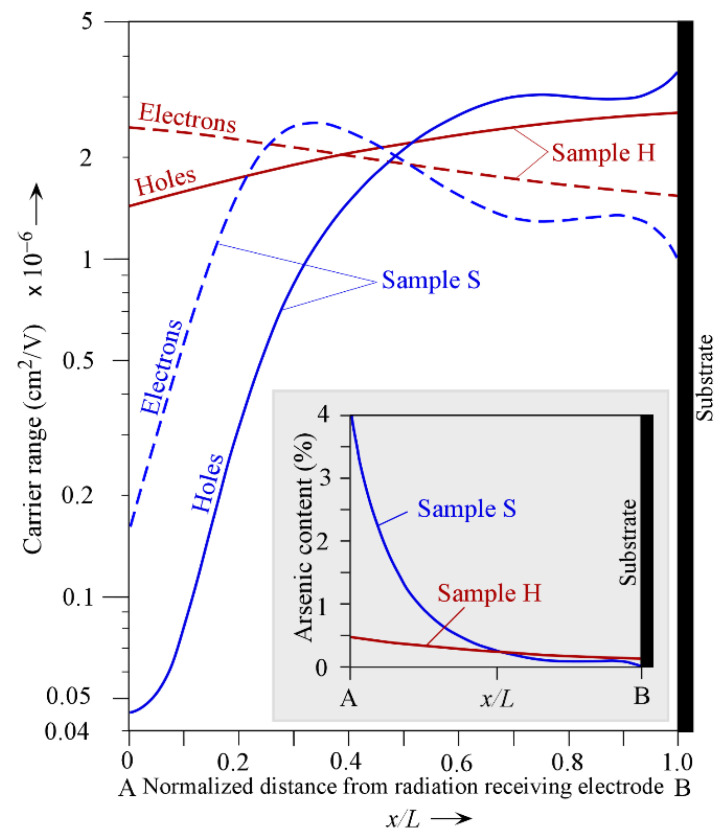
A semilogarithmic plot of the variation in the electron and holes ranges with normalized distance *x*/*L* from the radiation-receiving electrode (towards the substrate) for the two samples H and S in Figure 1.

The CE model requires the spatial dependence of both the electron and hole ranges *r_e_*(*x*) and *r_h_*(*x*), respectively, as described in Section 2.4. The spatial variation in the As content in Equation (1) was substituted into Equations (2) and (3) to obtain *r_h_*(*x*) and *r_e_*(*x*) variation across the thickness of the film, as shown in Figure 6. It can be seen that, in sample H, *r_h_*(*x*) increases monotonically and *r_e_*(*x*) decreases monotonically. The situation is quite different for sample S, which is the near-worst-case fractionation. The electron range *r_e_*(*x*) exhibits a large variation, more than an order of magnitude, with two maxima and one minimum. On the other hand, the hole range *r_h_*(*x*) changes by two orders of magnitude and has a broad minimum near the substrate. It will be shown in Section 3 that such large variations have a large impact on the hole and electron CEs.

### 2.4. Charge Collection Efficiency

**Figure 7:**
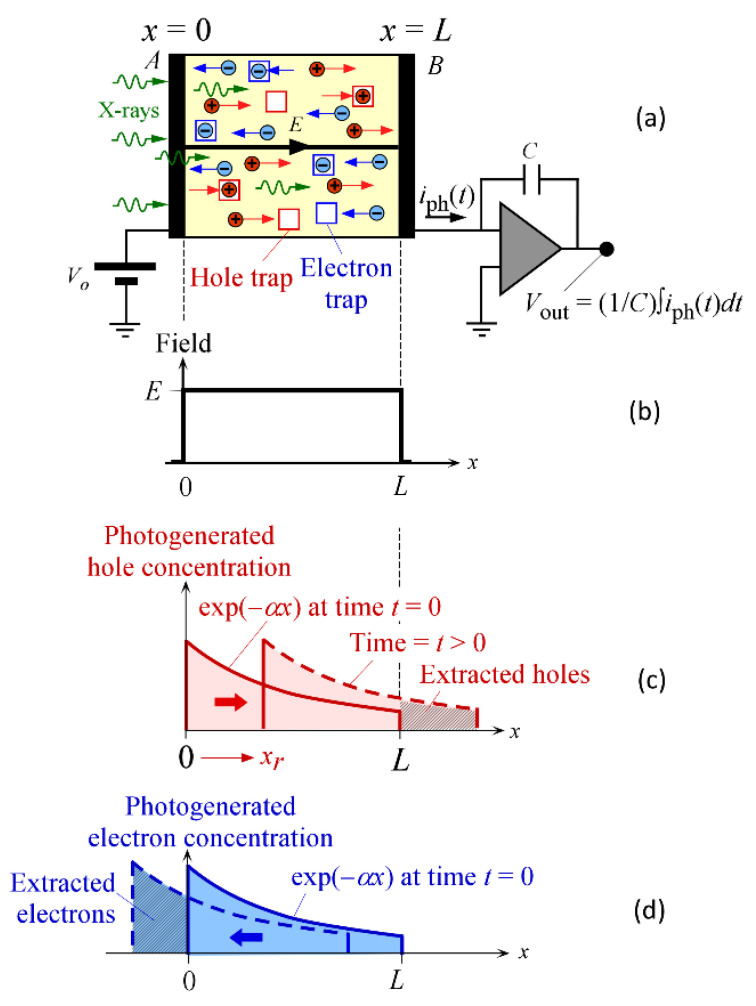
(**a**) A high-resistivity semiconductor used in an integrating detector. The incident radiation (X-rays) photogenerates electrons and holes which drift. Some of the carriers are captured in deep traps. (**b**) The field is assumed to be uniform. (**c**) The hole photogeneration profile at *t* = 0 and some time later. (**d**) The electron photogeneration profile at *t* = 0 and some time later.

Consider an integrating detector that uses a semiconductor with high resistivity (such as a-Se), as shown in Figure 7a. A time-of-flight (TOF)-type detection is assumed, in which the incident radiation is a very short pulse (a *δ*-function) of X-rays that generates a photocurrent *i*_ph_(*t*). The latter is integrated to obtain the signal *Q_c_*, the collected charge. The technique and the analysis have recently been reviewed in [17]. The X-ray-generated charge is called the injected charge *Q_i_*. The ratio *Q_c_*/*Q_i_* is the CE *η_c_*. Under small signals and in the absence of any pretrapped charges, one can assume that the perturbation to the field *E* is negligible, and *E* is uniform, as in Figure 7b. If the CE is independent of the intensity of the radiation, then one can readily apply the linear system theory and calculate the collected charge for an input radiation of any time dependence. Incident radiation is assumed to be single-energy X-rays with a photon energy *ε*. The photon fluence in the medium decays exponentially as exp(−*αx*), where *α* is the linear attenuation coefficient which depends on *ε*. (The normal notation for the linear attenuation coefficient is *μ*, but this is used to denote drift mobility in this paper.) The photogeneration of holes and electrons essentially follows this exponential profile at *t* = 0, as shown in Figure 7c,d. The electrons and holes drift in opposite directions and the ensuing photocurrent flows until all slowly drifting carriers are collected—electrons in the case of a-Se.

If we neglect the recombination of injected electrons and holes (a reasonable assumption under small signals) and assume deep trapping only with well-defined carrier ranges that are uniform across the sample thickness, then the CE, *η_co_*, can be written as the sum of the hole and electron collection efficiencies, HCE and ECE, respectively, as follows [18]:

(4) $$\begin{array}{l} \eta_{co}^{}=\mathrm{HCE}+\mathrm{ECE}=\frac{r_{h}E}{L}\left(1-\frac{\left[\exp\left(-\frac{L}{r_{h}E}\right)-\exp\left(-\frac{L}{\delta }\right)\right]}{\left[1-\exp\left(-\frac{L}{\delta }\right)\right]\left[1-\frac{\delta }{r_{h}E}\right]}\right)\\ +\frac{r_{e}E}{L}\left(1-\frac{\left[1-\exp\left(-\frac{L}{\delta }-\frac{L}{r_{e}E}\right)\right]}{\left[1-\exp\left(-\frac{L}{\delta }\right)\right]\left[1+\frac{\delta }{r_{e}E}\right]}\right) \end{array}$$

where *δ* is the attenuation depth (*δ* = 1/*α*). Equation (4) has been one of the most useful equations in the evaluation of various photoconductors for radiation detection and imaging applications, with numerous examples in the literature (see, e.g., [19,20,21]). It should be emphasized that the usefulness of Equation (4) is not limited to small signals. It can still be used under high radiation intensities with errors quantified in [22]. Equation (4) can also be used for a semiconductor in which the carrier range varies monotonically (either simply increasing or decreasing along *x*) if one defines appropriately averaged carrier ranges [6].

There are three definitions of carrier range of interest. The first is the range at the average composition, RAC, defined by:

(5) $$\mathrm{RAC}=\left\{\begin{array}{c} \mathrm{Equation}\;\left(2\right)\;\mathrm{for}\;\mathrm{holes}\;\mathrm{at}\;\bar{C}\\ \mathrm{Equation}\;\left(3\right)\;\mathrm{for}\;\mathrm{electrons}\;\mathrm{at}\;\bar{C} \end{array}\;\right.$$

The range from spatial averaging (RSA), $\bar{r}$, is defined by the simple spatial averaging of the range *r*(*x*) across the film thickness:

(6) $$\mathrm{RSA}=\bar{r}=\frac{1}{L}\int_{0}^{L}r(x)dx$$

The range from the average inverse, RAI, ${\bar{r}}_{\mathrm{inv}}$, is defined by:

(7) $$\frac{1}{\mathrm{RAI}}=\frac{1}{{\bar{r}}_{\mathrm{inv}}}=\frac{1}{L}\int_{0}^{L}\frac{dx}{r(x)}\;$$

Thus, there are six ranges to consider: three for holes, HRAC, HRSA, and HRAI, and three for electrons, ERAC, ERSA, and ERAI, where the first letter indicates whether it is a hole or electron range. All six ranges are used to calculate the corresponding hole and electron CEs from Equation (4) to test whether the concept of an “average range” (with a suitable definition of “average”) can still be used in the present case (in particular for sample S).

The formalism of the calculation of the CE *η_c_* in the presence of spatially varying carrier ranges has been formulated in [18,23] with the result that:

(8) $$\begin{array}{l} \eta_{c}=\mathrm{HCE}+\mathrm{ECE}=\frac{\alpha }{L[1-\exp(-\alpha L)]}\int_{0}^{L}\exp(-\alpha x)dx\int_{x}^{L}\exp\left\{-\int_{x}^{x^{\prime }}\frac{dx^{\prime \prime }}{r_{h}(x^{\prime \prime })E}\right\}\;dx^{\prime }\\ +\frac{\alpha }{L[1-\exp(-\alpha L)]}\int_{0}^{L}\exp(-\alpha (L-z))dz\int_{z}^{L}\exp\left\{-\int_{z}^{z^{\prime }}\frac{dz^{\prime \prime }}{r_{e}(z^{\prime \prime })E}\right\}\;dz^{\prime } \end{array}$$

in which the radiation-receiving electrode is positively biased. If the radiation-receiving electrode is biased negatively, then the hole and electron ranges need to be interchanged, since they would be drifting in opposite directions to those shown in Figure 7a,c,d. A derivation based on free carrier dynamics and the assumptions behind Equation (8) are discussed in [6]. In this work, Equation (8) is integrated numerically with the electron and hole ranges *r_e_*(*x*) and *r_h_*(*x*), varying with the distance from the radiation receiving electrode, as given in Figure 6. The numerical integration was carried out using a mathematics software called LiveMath (formerly, Theorist) with an accuracy to at least six decimals. Both positive and negative biases are considered with the field varying from some small value 0.1 V/μm to its maximum value 10 V/μm, as used in commercial X-ray detectors.

The a-Se detector is assumed to have a thickness *L* = 200 μm and the carrier ranges shown in Figure 6. The field range *E* = 1 to 10 V/μm corresponds to a bias variation of 200 V to 2 kV. Single-energy X-rays at *ε* = 20 keV and *ε* = 30 keV are considered, corresponding roughly to the average energy and the upper-end energy of the mammographic spectrum. Table 2 summarizes the values assigned to various quantities used in CE calculations.

## 3. Results and Discussion

**Figure 8:**
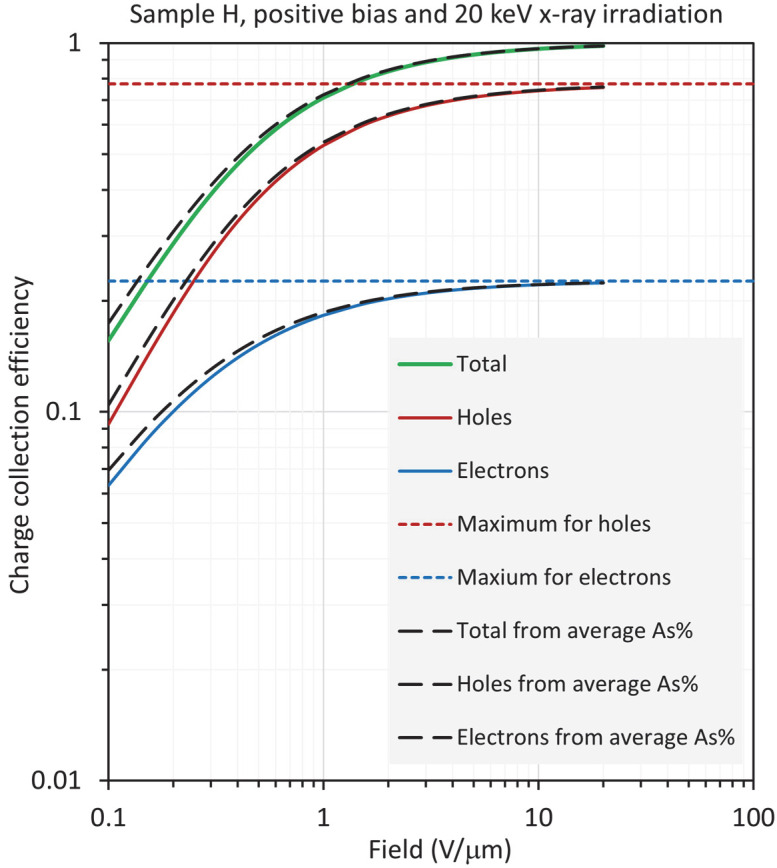
Log–log plot of the CE as a function of field for sample H (near optimum), biased positively and exposed to X-rays with a photon energy of 20 keV, so that *δ*/*L* =0.243.

**Figure 9:**
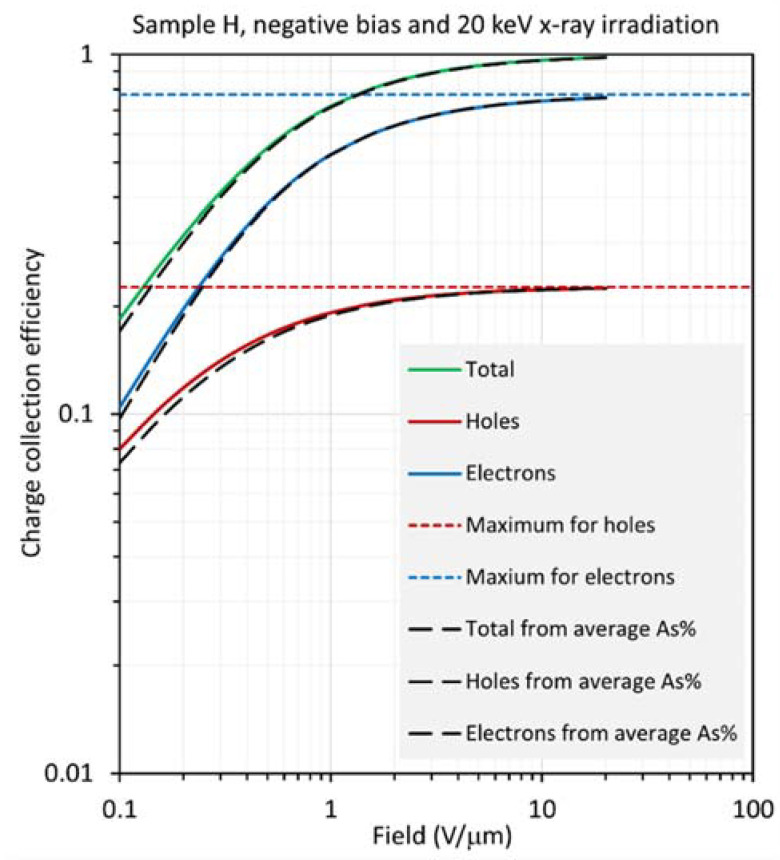
Log–log plot of the CE as a function of field for sample H (near optimum), biased negatively and exposed to X-rays with a photon energy of 20 keV, so that *δ*/*L* =0.243.

Equation (8) for positive bias was integrated numerically for an a-Se photoconductive layer, which is biased from an applied field of 1 V/μm to 20 V/μm using the spatial dependence of the electron and hole ranges in Figure 6 for both samples, H and S, at X-ray energies of 20 keV and 30 keV. Figure 8 and Figure 9 show the results for sample H biased positively and negatively, and exposed to X-rays with a photon energy of 20 keV. The HCE and ECE and the total CE (the sum of the latter two) were also calculated using the average composition $\bar{C}$ (= 0.257%), HRAC (*r_h_*_av_), and ERAC (*r_e_*_av_) (see Table 2) in the standard equation in Equation (4). These are shown as dashed lines in Figure 8 and Figure 9. It is clear that the CE calculations based on an average composition are almost identical to the actual CE values from Equation (8); any deviation, which is small, is at the lowest fields. Thus, the mean composition approach provides a reliable method for the calculation of the CE in samples with optimum fractionation, as in sample H. One only needs the average composition of the film and use of the corresponding carrier ranges from Equation (5).

Nonetheless, there are very small deviations at the lowest fields in Figure 8 and Figure 9. In Figure 8, under positive bias, both HCE and ECE are slightly overestimated with the result that the total CE is slightly overestimated, although the main contribution comes from the holes. The reverse arguments apply under negative bias in Figure 9.

When the calculations are repeated for X-rays with a photon energy of 30 keV, even better agreement was observed between the actual CE and that from the average composition. This is not unexpected, since the carriers are generated within ~0.73*L*, that is, more of the volume around the mean composition (*x* = 0.445*L*) of the film is used. One can therefore conclude that, for a-Se films with a slowly varying As composition across the film thickness, it is sufficient to use the carrier ranges *r_h_*_av_ and *r_e_*_av_, corresponding to ranges at $\bar{C}$, to calculate the CE based on Equation (4).

**Figure 10:**
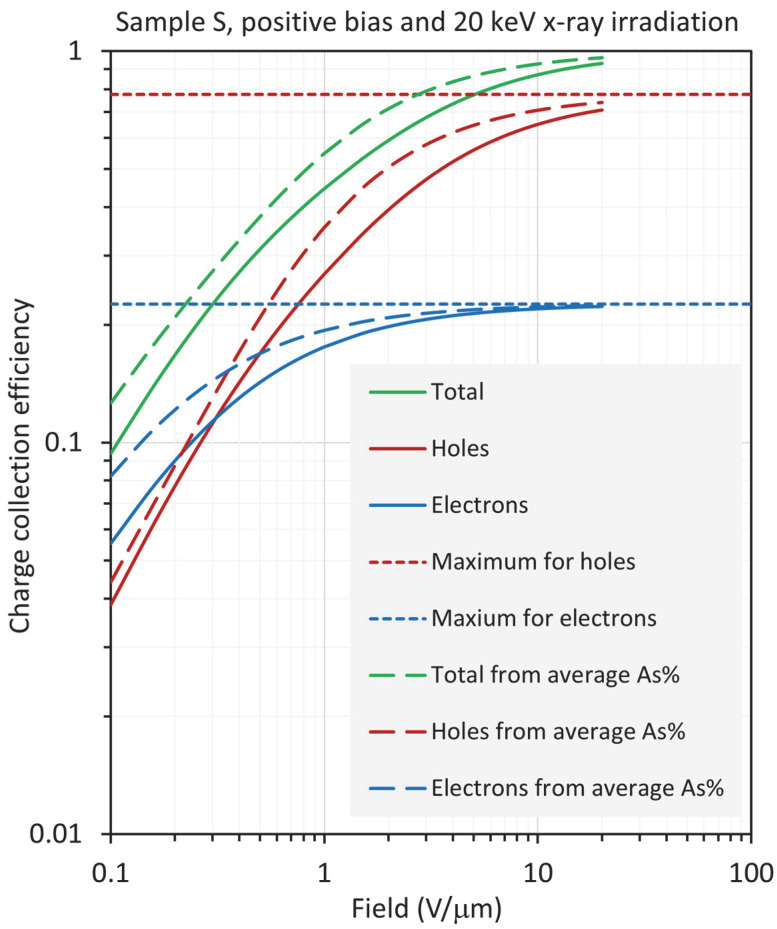
Log–log plot of the CE as a function of field for sample S (near worst case), biased positively and exposed to X-rays with a photon energy of 20 keV, so that *δ*/*L* =0.243.

**Figure 11:**
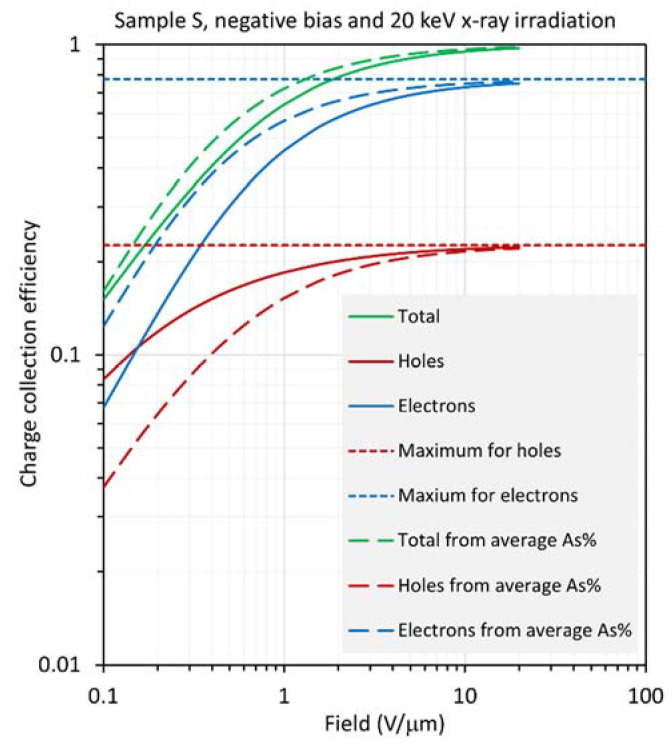
Log–log plot of the CE as a function of field for sample S (near worst case), biased negatively and exposed to X-rays with a photon energy of 20 keV, so that *δ*/*L* =0.243.

**Figure 12:**
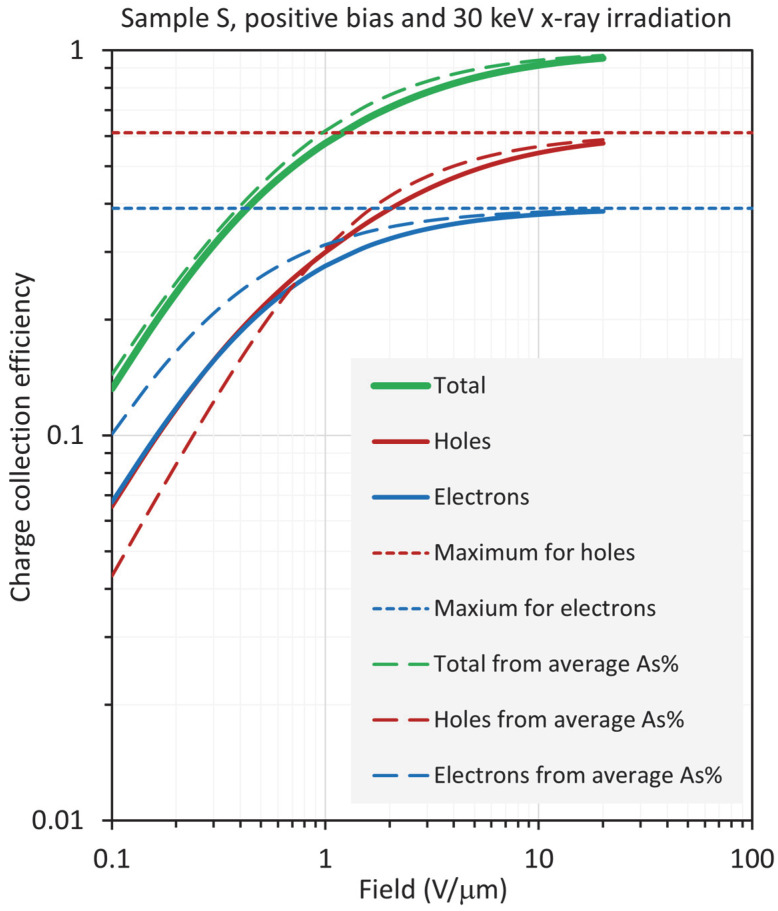
Log–log plot of the CE as a function of field for sample S (near worst case), biased positively and exposed to X-rays with a photon energy of 30 keV, so that *δ*/*L* =0.73.

**Figure 13:**
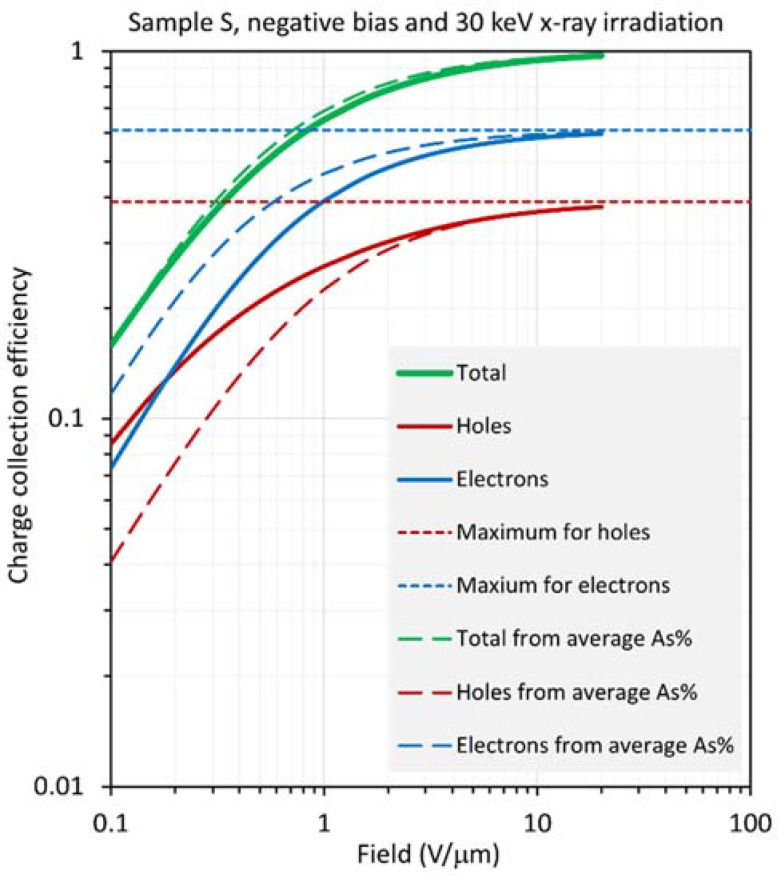
Log–log plot of the CE as a function of field for sample S (near worst case), biased negatively and exposed to X-rays with a photon energy of 30 keV, so that *δ*/*L* =0.73.

The results for sample S are distinctly different from those for sample H, in that large differences are observed between the CE calculated from the RAC and the actual CE, especially at low fields. The results are shown in Figure 10, Figure 11, Figure 12 and Figure 13 for positive and negative bias and for X-ray exposure at 20 keV and 30 keV. First, consider the results under 20 keV irradiation, which corresponds to *δ* = 0.24*L*, which is about 80% of the location of the average composition (0.303*L*). Figure 10 and Figure 11 clearly indicate that, compared with the actual CEs, (a) under positive bias, both HCE and ECE are overestimated and, consequently, the total CE from the RAC is also overestimated by a large amount; (b) under negative bias, ECE is overestimated but HCE is underestimated, with the result that the total CE from the RAC is only slightly overestimated.

The results in the case of 30 keV radiation are surprising in the sense that the total CE from the RAC is very close to the actual total CE, as shown in Figure 12 and Figure 13 for positive and negative bias, respectively. Under positive bias, the ECE from RAC overestimates the actual ECE. The HCE from RAC, on the other hand, underestimates the actual HCE below ~1 V/μm and overestimates it above ~1 V/μm. The latter two, then, sum to an overall CE that is very close to the actual value, as shown in Figure 12. The reason for this is that, at 30 keV, the penetration depth *δ* = 0.73*L*, whereas the average composition is at 0.303*L*. Thus, the photogeneration under 30 keV radiation uses a much higher volume of the film than the radiation at 20 keV. Under negative bias, it can be seen that, even though the HCE and ECE from RAC under- and overestimate the actual CE, respectively, the sum of the latter two is almost identical to the actual CE.

It is of interest to evaluate the errors involved in CE calculations at a particular field using the RAC, and the ranges from the spatial average, RSA, and average inverse, RAI, i.e., the range values in Table 2. The CE calculations were carried out at 5 V/μm and the errors in CE were evaluated based on different definitions of the average range, as listed in Table 3. Only sample S is considered. For example, the error involved in calculating the HCE by using the RAC, *r_h_*_av_, is defined by:

(9) $$\mathrm{Error}\;\mathrm{in}\;\mathrm{HCE}\;\mathrm{with}\;\mathrm{RAC}\left(\%\right)=100\left[\frac{\mathrm{First}\;\mathrm{term}\;\mathrm{in}\;\mathrm{Equation}\;\left(4\right)\;\mathrm{with}\;\mathrm{HRAC}-\mathrm{First}\;\mathrm{term}\;\mathrm{in}\;\mathrm{Equation}\;\left(8\right)\;\mathrm{with}\;\mathrm{HRAC}}{\mathrm{First}\;\mathrm{term}\;\mathrm{in}\;\mathrm{Equation}\;\left(8\right)\;\mathrm{with}\;\mathrm{HRAC}}\right]$$

and similar definitions for HCEs and ECEs, based on using averages in Equations (5)–(7). The results are summarized in Table 3 for positive and negative bias for X-rays at 20 and 30 keV.

The examination of Table 3 shows that, under positive bias, the lowest error for HCE comes from using the HRAI, that is, the average of the inverse hole range. According to the CE calculations in [6], if the hole range increases in the direction of drift of photoinjected holes, as in the present case (see Figure 6), then the range to use in Equation (4) is that from Equation (7), HRAI. This is indeed the result in the present case for 20 keV radiation with an error of −11.3%, but the errors from using HRSA and HRAI are comparable under 30 keV radiation. The minimum error for ECE is obtained by using the ERSA. In the direction of drift of photoinjected electrons, the electron range exhibits an overall tendency to decrease. In the latter case, the correct range to use is the ERSA. The errors are 0.17% and 0.11% at 20 keV and 30 keV radiation, respectively, so are negligibly small.

Under negative bias, the drift directions of holes and electrons are reversed, and we would expect HRSA and ERAI to yield the least errors in the HCE and ECE calculations, respectively. As can be seen from Table 3, HRSA provides the least error in HCE, whereas ERSA provides the least error in ECE, in contrast to expectations. The reason for this is that the electron range in sample S is not a simple monotonically changing function; it has two maxima and one minimum. However, the difference between the errors in using ERSA and ERAI is not large.

It is important to highlight that, normally, the composition profile *C*(*x*), and hence the hole- and electron-range profiles, will not be available. The average As content $\bar{C}$ in the film can be determined using various techniques, and this $\bar{C}$ is therefore the only quantity available for CE calculations. As can be seen from Table 3, the total CE calculated from the use of $\bar{C}$ has errors that are under 12% in magnitude; the worst case is for the total CE from RAC, which is 11.9%. The individual CEs, on the other hand, can have significant errors. For example, under negative bias and 30 keV radiation, the error in ECE from $\bar{C}$ is 18.7%. The error in HCE from $\bar{C}$ under positive bias and 20 keV radiation is 15.6% in magnitude. Furthermore, as is apparent from Table 3 and Figure 8, Figure 9, Figure 10, Figure 11, Figure 12 and Figure 13, the intuitive notion that we should be able to calculate the total CE from RAC works reasonably well, even though the individual CEs (HCE and ECE) may not have the desired accuracy.

The above discussion has neglected the effect of any Cl doping. Generally, Cl in the ppm amounts greatly enhances the hole range and extinguishes the electron range [2]. The main problem in analyzing the ranges in Cl-doped a-Se:As alloy films is that we do not know the Cl profile in the film. A simple assumption of uniform CL doping across the film thickness would involve shifting hole-range characteristics up and electron-range characteristics down in Figure 6, i.e., HCE is enhanced while ECE is decreased.

The actual bias polarity of choice on the radiation-receiving electrode depends on the relative importance of HCE and ECE. Since the hole range is typically longer than the electron range in most commercial a-Se photoconductive films, positive polarity has been the primary choice on the radiation receiving electrode, particularly for Cl-doped a-Se layers.

It should be mentioned that the X-ray sensitivity *S*_x_, which refers to the photogenerated charges collected per unit incident radiation and per unit area, depends on the following three factors: (1) the amount of X-ray radiation that is absorbed, which depends on the linear attenuation coefficient, *α*, the energy absorption coefficient of the medium, *α*_en_, and *L*. (2) The conversion of the absorbed radiation to charge carriers, which depends on the ionization energy of the medium, *W_i_*. (3) The charge collection efficiency, CE. If Φ is the incident X-ray fluence (photons per unit area per gray in air), then *S*_x_ as total charge collected per unit area per gray in air is given by:

(10) $$S_{x}=\left\{\Phi \left[\frac{\alpha_{\mathrm{en}}\left(\varepsilon \right)}{\alpha \left(\varepsilon \right)}\right]\left[1-\exp\left(-\alpha \left(\varepsilon \right)L\right)\right]\right\}\left[\frac{\varepsilon }{W_{i}\left(\varepsilon ,E\right)}\right]\mathrm{CE}\left(\varepsilon ,E\right)\;$$

where “(*ε*, *E*)” represents the dependence on the photon energy *ε* and applied field *E*. It would be reasonable to assume that the changes in *α*, *α*_en_, and *W_i_* with the As composition across the a-Se film are small, so that the effect of fractionation on *S*_x_ would be determined primarily by the changes in the CE in Equation (10).

Finally, it is useful to comment on the effect of the absorbed radiation on the charge carrier ranges in a-Se. As shown previously [24], the irradiation of a-Se with X-rays generates deep hole traps and hence shortens the hole lifetime and the range, while the electron transport seems to remain relatively unaffected. The present work assumes that there are no X-ray-generated deep traps in the sample, that is, the sample has been rested for a sufficient period of time for X-ray-induced defects to anneal out. In addition, the incident radiation is assumed to be sufficiently weak, so that the population of new defects generated and the subsequent changes in the lifetime are negligibly small compared with the changes in the lifetime across the film due to fractionation effects.

## 4. Summary and Conclusions

Fractionation effects during the vacuum deposition of a-Se photoconductive layers leads to a variation in As composition across the thickness of the film. Two cases were considered, both taken from the literature. The first corresponds to a near-optimum profile in which the As content decreases very slowly across the film thickness from the radiation-receiving electrode to the substrate, and is represented by a quadratic dependence (Figure 2). The second case is the near-worst-case profile in which there is a large change in the As content across the film thickness, which is represented by a fifth-order polynomial (Figure 3). Published data on electron and hole ranges in a-Se_1−*x*_As*_x_* alloys were extracted to construct a unified dependence of the carrier ranges on the As content. While the hole range decreases exponentially with the As composition (Figure 4), the electron range exhibits a maximum around ~0.7%As and then drops with the As content (Figure 5). The spatial dependences of the electron and hole ranges were then obtained in the two samples (Figure 6). The spatial dependence of the hole and electron ranges were then used in the total charge collection efficiency (CE) equation, which is valid in the presence of spatially varying carrier ranges (Equation (8)). The latter provided the actual CE values. The ranges corresponding to the average composition in each film were used in the standard equation for the CE (Equation (4)), which assumes uniform carrier ranges. The results show that, for films in which the As composition gradient is small, the CE from the use of the average composition agrees well with the actual CE. In the film in which the As composition changes are large, the near-worst-case scenario, unusual characteristics for the dependence of the hole and electron CE on the applied field were observed for exposure to X-rays with energies of 20 keV and 30 keV. The errors involved in using a spatially averaged range and a range based on the average inverse were also investigated. Since normally the As composition profile is not available, one has to rely on using the average composition in the film. It is shown that the latter approach can be used under positive and negative bias and under 20 keV and 30 keV radiation with small errors, as quantified in this work.

## Figures and Tables

**Table 1:** Fractionation characteristics of a-Se films S and H in Figure 2 and Figure 3.

Property	Sample H (Near-Optimum Case)	Sample S (Near-Worst Case)	Comment
As in the source material, %	0.34	0.50	Normally, the As content is uniform in vitreous shots
Average As content, $\bar{C}$, %	0.257	0.685	Average from integrating *C*(*X*) over the sample thickness
RMSD of As content profile, %	0.092	0.904	Root mean square deviation (RMSD) from the mean for a function
Location of $\bar{C}$ *x*/*L*	0.445	0.303	Position of the average composition from A
As variation with *X* = *x*/*L*	First order: *C*_0_ = 0.4145; *C*_1_ = −0.3157; *R*^2^ = 0.66202nd order: *C*_0_ = 0.44495; *C*_1_ = −0.5281; *C*_2_ = 0.214; *R*^2^ = 0.6823	5th order: *C*_0_ = 3.9132; *C*_1_ = −23.696; *C*_2_ = 69.338; *C*_3_ = −112.49; *C*_4_ = 94.25; *C*_5_ = −31.32; *R*^2^ = 0.9976	Equation (1). There is more data scatter around *C*(*X*) in Hordon, 1989

**Table 2:** Values for various quantities in the CE model.

Property	Value	Comment
Density, *ρ*, g cm^−3^	4.28	Relaxed state. a-Se exhibits typical glass relaxation characteristics
Thickness, *L*, μm	200	a-Se thickness
Electron and hole ranges	Figure 6	Empirical
Applied field, *E*, V/μm	1–10	*E* = *V_o_* /*L*; *V_o_* is applied voltage
Magnitude of *V_o_*, kV	0.2 to 2	Positive and negative voltages
X-ray energy, *ε*, keV	20 and 30	Mammographic range
X-ray mass linear attenuation coefficient (*α*/*ρ*), cm^2^/g	48.18 (20 keV); 15.96 (30 keV)	NIST website. *ρ* is the density of a-Se
X-ray attenuation depth, *δ* = 1/*α*, μm	48.5 (20 keV); 146 (30 keV)	
*δ*/*L*	0.243 (20 keV); 0.732 (30 keV)	Normalized attenuation depth
Quantum efficiency, QE	0.984 (20 keV); 0.745 (30 keV)	QE as defined in medical physics: fraction of attenuated X-ray photons
Sample H		
HRAC, *r*_*h*av_,*r*_*h*_ at $\bar{C}$, cm^2^/V	1.968 × 10^−6^	Equation (5)
HRSA, ${\bar{r}}_{h},\;$ cm^2^/V	2.148 × 10^−6^	Equation (6)
HRAI, ${\bar{r}}_{h\mathrm{inv}}$, cm^2^/V	2.073 × 10^−6^	Equation (7)
ERAC, *r*_*e*av_ at $\bar{C}$, cm^2^/V	1.968 × 10^−6^	Equation (5)
ERSA, ${\bar{r}}_{e},\;$cm^2^/V	1.940 × 10^−6^	Equation (6)
ERAI, ${\bar{r}}_{e\mathrm{inv}}$, cm^2^/V	1.900 × 10^−6^	Equation (7)
Sample S		
HRAC, *r*_*h*av_, *r*_*h*_ at $\bar{C}$, cm^2^/V	0.884 × 10^−6^	Equation (5)
HRSA, ${\bar{r}}_{h},\;$ cm^2^/V	1.841 × 10^−6^	Equation (6)
HRAI, ${\bar{r}}_{h\mathrm{inv}}$, cm^2^/V	0.340 × 10^−6^	Equation (7)
ERAC, *r*_*e*av_, at $\bar{C}$, cm^2^/V	2.513 × 10^−6^	Equation (5)
ERSA, ${\bar{r}}_{e},\;$cm^2^/V	1.527 × 10^−6^	Equation (6)
ERAI, ${\bar{r}}_{e\mathrm{inv}}$, cm^2^/V	1.066 × 10^−6^	Equation (7)

The QE definition is that used in medical physics and not solid-state physics. SA: spatial or simple average; AI is the average of inverse range; $\bar{C}$ is the average As content in percent in the film.

**Table 3:** Errors involved in the calculation of the CEs using various definitions of carrier ranges for sample S.

Field Polarity	P	P	N	N
X-ray photon energy (keV)	20	30	20	30
Error in CE from RAC, %	11.9	4.95	3.25	5.83
Error in HCE from HRAC, %	15.6	6.34	−3.54	−13.5
Error in HCE from HRSA, %	26.8	15.4	1.00	12.3
Error in HCE from HRAI, %	−11.3	−15.7	−15.4	−51.1
Error in ECE from ERAC, %	2.24	3.06	5.36	18.7
Error in ECE from ERSA, %	0.17	0.113	1.12	1.27
Error in ECE from ERAI, %	−2.03	−2.98	−3.29	−13.6

CEs at *E* = 5 V/μm, P is positive, and N is negative on the radiation receiving electrode.

## Data Availability

Not applicable.

## References

[1] Kasap S., Frey J.B., Belev G., Tousignant O., Mani H., Greenspan J., Laperriere L., Bubon O., Reznik A., DeCrescenzo G. (2011). Amorphous and Polycrystalline Photoconductors for Direct Conversion Flat Panel X-Ray Image Sensors. Sensors.

[2] Kasap S.O., Kasap S.O. (2022). Doped and Stabilized Amorphous Selenium Single and Multilayer Photoconductive Layers for X-Ray Imaging Detector Applications. Photoconductivity and Photoconductive Materials.

[3] Schottmiller J.C. (1975). Structure–property relationships in xerographic selenium-alloy films. J. Vac. Sci. Technol..

[4] Sigai A.G. (1975). Open boat evaporation of low-arsenic-selenium alloys. J. Vac. Sci. Technol..

[5] Hordon M.J., Carapella S.C. (1989). Effect of alloy surface crystallization on the control of arsenic fractionation in arsenic-doped selenium photoreceptor films. Proceedings of the Fourth International Symposium on Uses of Selenium and Tellurium.

[6] Kasap S.O., Kabir M.Z., Ramaswami K.O., Johanson R.E., Curry R.J. (2020). Charge collection efficiency in the presence of non-uniform carrier drift mobilities and lifetimes in photoconductive detectors, *J*. Appl. Phys..

[7] Güneş O., Koughia C., Curry R.J., Gholizadeh A.B., Belev G., Ramaswami K.O., Kasap S.O. (2019). Optical and electrical properties of alkaline-doped and As-alloyed amorphous selenium films. J. Mater. Sci. Mater. Electron..

[8] Schottmiller J., Tabak M., Lucovsky G. (1970). Ward, A. The effects of Valency on Transport Properties in Vitreous Binary Alloys of Selenium. J. Non-Cryst. Solids.

[9] Kasap S.O., Juhasz C. (1982). Charge Transport in Selenium Based Amorphous Xerographic Photoreceptors. Photogr. Sci. Eng..

[10] Tabak M.D., Hillegas W.J. (1972). Preparation and transport properties of vacuum evaporated selenium films. J. Vac. Sci. Technol..

[11] Kolomiets B.T., Lebedev E.A. (1966). Influence of Impurities on the carrier mobility in amorphous selenium. Sov. Phys. Solid State.

[12] Juhasz C., Vaezi-Nejad S.M., Kasap S.O. (1985). Electron and hole drift mobility amorphous selenium-based photoreceptors. J. Imag. Sci..

[13] Belev G., Kasap S.O. (2006). Reduction of the dark current in stabilized a-Se based X-ray detectors. J. Non-Cryst. Solids.

[14] Marshall J.M., Fisher F.D., Owen A.E. (1974). The electron Drift Mobility in Arsenic-Selenium Glasses. Phys. Stat. Solidi A.

[15] Borisova Z.U. (1982). Glassy Semiconductors.

[16] Belev G.S., Fogal B., Koughia K.V., Johanson R.E., Kasap S.O. (2003). Dependence of charge-carrier ranges in stabilized a-Se on preparation conditions and alloying. J. Mater. Sci. Mater. Electron..

[17] Kasap S.O., Kasap S.O. (2022). Time of Flight Transient Photoconductivity Technique. Photoconductivity and Photoconductive Materials.

[18] Zullinger H.R., Aitken D.W. (1968). Charge Collection Efficiencies for Lithium-Drifted Silicon and Germanium Detectors in the X-ray Energy Region. IEEE Trans. Nucl. Sci..

[19] Semeniuk O., Grynko O., Decrescenzo G., Juska G., Wang K., Reznik A. (2017). Characterization of polycrystalline lead oxide for application in direct conversion X-ray detectors. Sci. Rep..

[20] Grynko O., Reznik A., Kasap S.O. (2022). Progress in Lead Oxide X-ray Photoconductive Layers. Photoconductivity and Photoconductive Materials.

[21] Kabir M.Z., Kasap S.O. (2002). Charge collection and absorption-limited sensitivity of X-ray photoconductors: Applications to a-Se and HgI2. J. Appl. Phys..

[22] Ramaswami K.O., Johanson R.E., Kasap S.O. (2019). Charge collection efficiency in photoconductive detectors under small to large signals. J. Appl. Phys..

[23] Miller G.L., Gibson W.M. (1962). Charge collection in semiconductor detectors. Proceedings of Nuclear Electronics I.

[24] Kasap S.O., Yang J., Simonson B., Adeagbo E., Walornyj M., Believe G., Johanson R.E. (2020). Effects of X-ray irradiation on charge transport and charge collection efficiency in stabilized a-Se photoconductors. J. Appl. Phys..

